# Why Assembling Plant Genome Sequences Is So Challenging

**DOI:** 10.3390/biology1020439

**Published:** 2012-09-18

**Authors:** Manuel Gonzalo Claros, Rocío Bautista, Darío Guerrero-Fernández, Hicham Benzerki, Pedro Seoane, Noé Fernández-Pozo

**Affiliations:** 1 Department of Molecular Biology and Biochemistry, Faculty of Sciences, University of Malaga, 29071 Málaga, Spain; Email: bhicham538@gmail.com (H.B.); seoanezonjic@uma.es (P.S.); noefp@uma.es (N.F.-P.); 2 Bioinformatics Andalusian Platform, Bio-innovation Building, University of Malaga, 29590 Málaga, Spain; Email: rociobm@uma.es (R.B.); dariogf@uma.es (D.G.-F.)

**Keywords:** plant sequencing, NGS, complexity, repeats, assemblers, polyploidy, bioinformatics

## Abstract

In spite of the biological and economic importance of plants, relatively few plant species have been sequenced. Only the genome sequence of plants with relatively small genomes, most of them angiosperms, in particular eudicots, has been determined. The arrival of next-generation sequencing technologies has allowed the rapid and efficient development of new genomic resources for non-model or orphan plant species. But the sequencing pace of plants is far from that of animals and microorganisms. This review focuses on the typical challenges of plant genomes that can explain why plant genomics is less developed than animal genomics. Explanations about the impact of some confounding factors emerging from the nature of plant genomes are given. As a result of these challenges and confounding factors, the correct assembly and annotation of plant genomes is hindered, genome drafts are produced, and advances in plant genomics are delayed.

## 1. Introduction

Higher plants are the Earth’s dominant vegetation in nearly all ecosystems. They sustain living beings (including humans) by providing oxygen, food, fiber, fuel, medicines, spirits, erosion defense, flooding control, soil regeneration, (bio)remediation, urban cooling, green spaces (including gardens) and CO_2_ lowering, and contributing to the control of global warming [1]. Higher plants also exhibit a wide range of forms, with individuals ranging in size from floating *Wolffia* plants of 1 mm in length to trees of more than 100 m in height or with a trunk diameter exceeding 10 m (such as the angiosperm *Eucaliptus regnans* and the gymnosperms *Sequoia sempervirens* and *Taxodium mucronatum*). Plants also contain the longest-living organisms (with *Pinus longaeva*, *Taxus baccata* and *Picea abies* individuals living on Earth for nearly 5,000–8,000 years). Moreover, plants are stuck in place and cannot escape enemies or uncomfortable conditions and need to develop strategies that improve their chances of survival due to sessility. So, plants have evolved tens of thousands of chemical compounds which they use to ward off competition from other plants, to fight infections, and to respond generally to the environment [2]. In consequence, plant species have larger and more complex genome sizes and structures than animal species and exhibit tremendous diversity in both size and structure [3]. Therefore, plants seem to be an important source of biological knowledge and economic profit, but relatively few plant species have been sequenced. In fact, in a world with >370,000 known plant species (with probably many thousands more still unclassified), only ~80,000 species have at least one single sequence in GenBank.

The publication of the first plant genome sequence of *Arabidopsis thaliana* [4] provided and improved the genetic landscape for studying all plants and has paved the way for sequencing several other plant genomes. It has also transformed the methods and tools for plant research and crop improvement [5]. *Arabidopsis*, and later *Oryza sativa* (rice) [6], *Carica papaya* (papaya) [7] and *Zea mays* (maize) [8] were sequenced using the classical Sanger method. The arrival of next-generation sequencing (NGS) technologies has allowed the rapid and efficient development of genomic resources for non-model or orphan plant species [9,10,11,12,13]. However, only *Arabidopsis* and rice—sequenced by Sanger’s method using a BAC-by-BAC approach—have been really finished to date, the rest being drafts in a greater or lesser stage of completion. Unfortunately, even the complete or gold standard genomes contain gaps in their sequences corresponding to highly repetitive sequences, which are recalcitrant to sequencing and assembly methods [14]. A summary of all published plant genome sequences to date can be found in Table 3 in [14] and in Table 3 in [5]. 

Since there is no central focus in the scientific plant world, the choice of plant genomes for sequencing has been driven mainly by cost efficiency and the avoidance of complexity, and hence only plants with relatively small genomes (median size of 466 Mbp) were selected for sequencing in the first instance, although the most important crops have a median size of 766 Mbp [5]. In fact, *Arabidopsis thaliana* proves to be an outlier amongst plants because its genome has undergone a 30% reduction in genome size and at least nine rearrangements in the short time since its divergence to *Arabidopsis lyrata* [1,15]. In many plant species, it is now clear that a single genome sequence does not necessarily reflect the entire genetic complement [16,17], opening a new branch in the study of pan‑genomes and core genomes [18].

Most plant sequencing efforts have been dedicated to angiosperms, mainly the eudicots, under which the most economically important crops are classified [19,20]. But sequencing efforts should be expanded beyond the traditional commodity crops and include other non-commodity crops and non‑model species (e.g., conifers, ferns and other bryophytes). We present here the current state of the art of challenges and confounding factors that explain why plant genomics is less developed than animal genomics and remains so focused on small genomes. We also discuss why challenges are not overcome by the arrival of NGS.

## 2. From Sanger Technology to NGS: Getting Plants off the Ground

While extremely successful in the past, Sanger sequencing [21] does present the following drawbacks for actual sequencing projects: (1) requirement of nucleic acid subcloning, (2) clone amplification in hosts, (3) low throughput, (4) slow sequencing speed, and (5) high costs (both in terms of consumables and salaries, averaging $1,330 per Mbp [22]). This is the reason why sequencing projects with Sanger technology have always been carried out by international consortia [4,8,23,24].

NGS strategies allow a single template molecule to be directly used to generate millions of bases at low cost with a less cumbersome laboratory protocol. There are three NGS platforms widely used nowadays that are considered to be second-generation sequencing: (1) the Genome Sequencer FLX+/454 from Roche which is capable of producing over a million reads of up to 800 bases per 10 hour run, yielding a total of 0.7–1 Gbp at a price of approximately $90 per megabase; (2) the Genome Analizer from Illumina, of which the latest version, HiSeq2000, yields 100 Gbp of bases per day (26–150 bp read length) at a cost of $4 per megabase; and (3) the Applied Biosystems SOLiD (Sequencing by Oligo Ligation and Detection) that produces 10–300 Gbp of short reads (up to 75 bp) per run at a similar cost. The three platforms offer the paired-end sequencing technique. As a result, even large plant genomes can count on relatively inexpensive deep coverage with reads of 100 bp and paired-end libraries from 1 to 5 kbp (we will see that deep coverage does not allow for complete plant sequencing). A detailed description is beyond the scope of this article, and several reviews illustrate the rapid evolution of these and the newest NGS technologies (to cite a few, [25,26,27,28,29,30,31]). While 454 FLX+ and Sanger technologies are considered to produce long reads (600–800 pb in average), the other two produce short reads (<150 bp in average). Short-read technologies compensate the shortness of the sequences with a high coverage, so that bacteria can be successfully sequenced with a 40×–50× coverage, but as the genome increases in complexity, coverage of 100× may still be inefficient [32,33,34]. In contrast, long-read technologies do not need such deep coverage, with 20×–30× being enough for a good compromise between costs and assembly quality [32].

NGS is becoming the new sequencing standard for the following reasons: (1) simplification of the sequencing process (DNA cloning is not required); (2) miniaturization and parallelization (low cost); and (3) good adaptation to a broad range of biological phenomena (genetic variation, RNA expression, protein-DNA interactions, gene capture, methylation, *etc.*). But not everything about NGS is an advantage [25]: (i) the base calls are at least tenfold less accurate than Sanger sequencing base calls; (ii) the sequence length is shorter than in Sanger technology and requires dedicated assembly algorithms; and (iii) the quality of the NGS assemblies is also lower than Sanger assemblies. As a result, most plant genomes sequenced by NGS produce “drafts” that are suitable for (1) establishing gene catalogues, (2) deciphering the repeat content, (3) glimpsing evolutionary mechanisms, and (4) performing early studies on comparative genomics and phylogeny. Unfortunately, drafts (i) hinder the progress of capturing accurately the information embedded in the repetitive fraction of the genome; (ii) make it difficult to distinguish genes from pseudogenes; and (iii) make it difficult to differentiate between alleles and even paralogues [35]. If only draft genomes are produced in the short future, plant genomics may face a crisis since, although the complex genomes of many more species are now accessible, the portion of each genome that can be reliably accessed has diminished substantially (<80%). The expertise and motivation for sequencing plant genomes to a high quality is disappearing, pushed by the rapid publication of a new draft genome lacking up to 20% of the genome [33].

Widespread adoption of NGS technology is tightly bound to bioinformatics. Integration of the many complex and rich sequencing datasets has yielded cohesive views of cellular activities and dynamics (for example, see [36,37,38]). The increase in plant sequence data has also prompted the development of dedicated repositories, such as the general purpose Phytozome [13], the comparative plant genomics resource PLAZA [39], plant family databases such as TreeGenes for forest tree genome data [40], or species specific databases (e.g., EuroPineDB for maritime pine [41], Eucawood for *Eucalyptus* [42], or MeloGen for *Cucumis melo* [43]). It is worth mentioning the iPlant project [44], which emerged with the aim of creating an innovative, comprehensive and foundational cyber infrastructure to support plant biology research, the VirtualPlant platform [45], integrating genome-wide data on the known and predicted relationships among genes, proteins, and molecules in order to enable scientists to visualize, integrate, and analyze genomic data from a systems biology perspective or the Plantagora platform [34], which addresses the gap between having the technical tools for plant genome sequencing and knowing precisely the best way to use them. 

NGS can be said to have accelerated biological research in plants by enabling the comprehensive analysis of genomes, transcriptomes and interactomes. Moreover, translational research has been spurred by NGS, the most successful case being the application of a gene from *A. thaliana* to improve abiotic stress tolerance traits in crops [5]. But if NGS only produces draft genomes, it could drive plant functional genomics into a dead end in the near future.

## 3. Challenging Features of Plant Genomes

Genome size, duplications and repeat content are factors to be considered for all genomes to be sequenced. In particular, plant genomes usually appear as gene islands among the background of high copy repeats (usually >80%), where 95% coverage of the genes is assumed, based on comparisons with cDNA databases. This discouraging situation can be explained by several plant features that hinder the sequence assembly and annotation, and severely limiting genomics research productivity.

### 3.1. Sampling

The main drawback of plant sequencing is that it is often very hard to extract large quantities of high-quality DNA from plant material, making it difficult to prepare proper libraries for sequencing. Additionally, although any genome sequencing project is afforded with samples from a single plant, the situation is completely different in transcriptome sequencing, where the traditional approach was to use a variety of tissues and conditions from different multiple accessions by different researchers, resulting in many extremely similar unigenes representing the same gene [41,42,43]. When such a heterogeneous transcriptome is studied using long reads, the presence of multiple alleles does not significantly hamper the unigene assembly [22], but when the transcriptome is studied with NGS technologies providing reads <100 bp, alleles and paralogues really do impair the assembly result.

### 3.2. Genome Size and Complexity

Plant-specific needs are sustained by new genes that may arise from gene duplications, alternative gene splicing, ploidy or gene retention following genome duplication, making plant genomes large and complex, as pointed out in the introduction. In fact, genome sizes across land plants range over two to three orders of magnitude, with an average around 6 Gbp, which is one order of magnitude larger than the average size of genomes sequenced so far [3]. Current sequencing technologies can manage large, complex genomes, such as wheat (*Triticum aestivum* with 16 Gbp split in 21 chromosomes) or pines (22–33 Gbp split in 12 chromosomes), so the genome size is not an unassailable issue anymore. The real problem is not the genome size *per se* but the complexity of the genomes, since the number of genes does not vary to the same extent as the genome size. The length of single-copy regions (always flanked by repeated sequences [12]) varies widely among plant species. In general, two types of arrangements are recognized: (1) short period interspersion (single copy sequences of 300–1,200 bp interspersed as islands among short lengths (50–2,000 nt) of repeat sequences); and (2) long period interspersion(single copy sequence islands of 2,000–6,000 bp interspersed among long repeat sequences). Genome size appears to be related to the type of interspersion: Plant species with small genomes, such as *Arabidopsis*, have long period interspersion and longer lengths of non repetitive sequences; on the contrary, plant species with large genomes, such as wheat, rye or maize, have short period interspersion and shorter non-repetitive sequences [46]. This confirms the intuitive notion that small genomes are therefore less difficult to assemble than larger genomes. The different factors that can contribute to the large variation of genome size and complexity in plants are discussed below.

### 3.3. Transposable Elements

During evolution, transposons have introduced profound changes in genome size, structure and function between species and within species [18], accounting for the major force in reshaping genomes [47]. This could explain why Chromosomes 1 and 2 of *A. thaliana* are a fusion of Chromosomes 1 and 2, and 3 and 4, respectively, of *A. lyrata* [15,47]. Transposable elements are by far the most highly represented repetitive sequence in plant genomics: due to the replicative nature of the retrotransposition process, Class I transposons (including retrotransposons) can account for up to 90% of all the transposons, while Class II elements are much less abundant [48]. Small-genome plants like *Arabidopsis* and rice are sparsely populated by transposons, containing 5.6% and 17% respectively. In contrast, the transposon-derived fraction of medium/large genomes may reach 85% in maize and >70% in barley [8,49,50]. Owing to their abundance and repetitive nature, transposable elements complicate genome assembly, particularly when short-read technologies are used [51].

### 3.4. Heterozygosity

Most plants are heterozygous, particularly those that have not been domesticated in laboratories [52]. Since it is a kind of redundancy, which is always a challenging factor in assemblies, only euchromatic regions of the genomes can be assembled, and a high percentage of NGS reads remain unassembled (15% in poplar [*Populus trichocarpa*] [53]). This happens even if a hierarchical clustering guided by a physical map is used to guide the sequence assembly. As a result, the poplar genome seems to contain a duplicated gene content since most loci present both possible alleles. The relative incompleteness of both heterozygous genomes demonstrates the difficulty of producing high‑quality genome sequences for a natural, heterozygous cultivar with current sequencing technologies. As a consequence, some plant-sequencing projects tend to focus on homozygous derivatives, even if they are not commercially or agronomically important. This was the case, for example, for the highly homozygous genotype of *Vitis vinifera* (grape) in 2007 [54]. Another problem introduced by heterozygosity is the creation of false segmental duplications in assemblies that occur when heterozygous sequences from two haplotypes are assembled into separate contigs and are scaffolded adjacent to each other rather than being merged [55]. In conclusion, only the use of longer reads would improve the ability to assemble separate haplotypes within a genome (see ‘Polyploidy’ section below).

### 3.5. Polyploidy

Polyploidy is the result of the fusion of two or more genomes within the same nucleus. It originates from either whole-genome doubling (autopolyploidy) or by interspecific or intergeneric hybridizations followed by chromosome doubling (allopolyploidy). Genome duplication has the following potential advantages: (i) it is a source of genes with new functions and new phenotypes, (ii) some polyploids appear to be better adapted as a consequence of genome plasticity [56], and (iii) others lose their self‑incompatibility, gain asexual reproduction, and produce higher levels of heterozygosity; this may explain the widespread occurrence of polyploids in plants [57]. Polyploidization is therefore one of the major driving forces in plant evolution and is extremely relevant to speciation and diversity [1,58]. An ancestral triplication affecting most (or perhaps all) dicots was followed by two additional whole‑genome duplications [1,15]. Every plant lineage shows traces of additional, independent and more recent whole genome duplications somewhere between 50 and 70 million years ago [15]. Some genes have been repeatedly restored to single-copy status following many different genome duplications [59], with the degree of gene retention differing substantially in the different taxa. Therefore, the resulting assembly of a plant genome is dependent on whether the species is an autopolyploid, an allopolyploid, or on the age of the ploidization event. Sequencing of recent polyploids will be especially complex depending on the divergence of the duplicated genes, particularly in the case of many important crops that are true polyploids (banana, potato, cotton, wheat or sugarcane). The redundancy created by the presence of two or more sets of genes within a nucleus can affect the accuracy of the assembly, and the need to differentiate between homologues could influence the final utility of the obtained sequence. Indeed, contigs can break at polymorphic regions or misassemblies can be obtained between large-scale duplications.

The ploidy issue has been ‘resolved’ in different ways. For example, since most cultivated potatoes are tetraploids, the Potato Genome Sequencing Consortium decided to use as reference a doubled monoploid that was homozygous for a single set of the 12 chromosomes [60]. The authors found that the two haplotypes within a heterozygous diploid were more divergent from each other than from the single haplotype used as reference. In the case of the cultivated strawberry, which is allo-octaploid, the diploid species *Fragaria vesca* (woodland strawberry) was sequenced to bypass the difficulties of polyploidy [61]. For hexaploid wheat, the Wheat Genome Initiative has decided to follow another strategy: a flow cytometry separation of the 10 chromosomes one by one or in groups, the construction of a tiling BAC physical map, and subsequent sequencing of each chromosome using a BAC-by-BAC strategy [8].

### 3.6. Gene Content and Gene Families

The gene content in plants can be very complex, as shown by the presence of large gene families and abundant pseudogenes derived from recent genome duplication events and transposon activity (see above and [8]). For example, there are remnants of chloroplast and mitochondrial genes in the nuclear genome that skew coverage levels [7], such as ~270 kbp of the mitochondrial genome inserted into Chromosome 2 of *Arabidopsis* [62]. But gene duplication is regarded as a major force in the origin of new genes and genetic functions. By way of example, the appearance of C4 photosynthesis has evolved from the C3 pathway and has appeared independently on at least 50 occasions during plant evolution [63]. Other examples of gene duplication are the striking increase in the number of starch‑associated genes in papaya (39) with respect to *Arabidopsis* (20), or the expanded number of kinase family members, cytochromes P450 and the enzymes engaged in plant secondary metabolism [64]. However, recent comparisons of *Arabidopsis*, poplar, grapevine, papaya and rice genomes estimated that the angiosperm ancestor should contain between 12,000 and 14,000 genes [15]. As a result, more than half of plant genes are really a gene family, 45% of them with the same function but different expression patterns [65]. Specific strategies are required to distinguish alleles from paralogues when sequencing natural heterozygous isolates, although this is not expected to have a very promising success in the near future [59]. Moreover, the presence of out-paralogues produced by duplication prior to the divergence of two lineages and in-paralogues produced in each lineage, together with the multiple rounds of polyploidy in plant lineages, accentuate these problems as divergence between paralogues occurs at different paces. 

A curious finding in virtually all eukaryotic genomes sequenced to date (including plants) is the existence of lineage-specific genes for which an orthologue cannot be discerned in closely related species. Lineage-specific genes are a tantalizing target for functional studies since they should distinguish closely related taxa, but unfortunately, these apparently ‘lineage-specific genes’ could simply be the result of misassemblies [1]. Attention should be paid to these genes before a promising theory can be proposed. Bioinformatic efforts should be made to distinguish real, new genes from misassembled sequences, since we suspect that apparently new genes in sequences <150 bp in length correspond to misassemblies [66]. This also explains the fact that gene sequences may not always be correct, since nearly identical gene families are notoriously difficult to assemble and may collapse into a mosaic sequence without necessarily representing any member of the family [67].

Finally, gene movements can affect plant genome assembly. Gene movement studies found that many gene categories in *Arabidopsis*, papaya and grape were recently transposed at a basal frequency of 5%. The most striking result was that some gene families exhibited very high movement frequencies (50%–90%) [1,68]. This should not be a problem for any assembly procedure since jumping usually occurred a long time ago and the sequences have diverged, but the real drawback is that the region around the transposed gene is enriched with authentic transposons, phantom transposons and pseudogenes [69]. This situation directly impinges on the problem of assembly of repeated sequences and can cause gene loss in the assembly due to collapse of the repetitive surroundings.

### 3.7. Non-Coding RNAs

Non-coding RNAs (ncRNAs) were first described in plants in 1993 [70] and since then, they have provided new insights into gene regulation in plant and animal systems. The advent of NGS has produced a profound impact on the discovery of new ncRNAs. There are small ncRNAs with mature lengths below 30 bp, such as microRNA (miRNA), small interfering RNA (siRNA) and Piwi‑interacting RNAs (piRNAs, usually found in animals). Long ncRNAs (200 bp long or more) are another subset of ncRNAs that contain many signatures of mRNAs, including 5' capping, splicing and poly-adenylation, but have little or no open reading frame [71]. Genomic sequences within ncRNAs are often shared within a number of different coding and non-coding transcripts in the sense and antisense directions giving rise to a complex hierarchy of overlapping isoforms. To add even more complexity to ncRNAs, a high proportion of them are variants of protein-coding cDNAs. When using short-read NGS strategies, sequence complexity frustrates the assembly of ncRNA precursors due to their repetitive nature since most ncRNAs contain fragments that are complementary to one or more genes, which causes the collapse of assemblers at the exon or, primarily, at the ncRNA gene [72]. Only long read-based strategies could cover both mature ncRNAs and ncRNA precursors provided that long ncRNAs are not longer than the read lengths.

### 3.8. Widely Distributed Repetitive Sequences (Low Complexity Sequences)

Plants share with other organisms a common source of general repetitive sequences [73] that are a source of low complexity regions, which are always a problem for assemblies. The main sources of repeats are the following:

- **Repetitions among chromosomes:** Duplications occurring both within chromosomes (e.g., ~250 tandem duplications each of ~10 kbp on Chromosome 2 of *Arabidopsis*) and between chromosomes (e.g., ~4 Mbp long regions between Chromosomes 2 and 4, or 700 Mbp long regions between Chromosomes 1 and 2 in *Arabidopsis*; ~3 Mbp at the termini of the short arms of Chromosomes 11 and 12 in rice, as well as Chromosomes 5 and 8 in sorghum) [62,74].
- **rDNA units:** These contain the rRNA genes, which are presented as hundreds of copies. Each unit is typically 10 kbp in plants and as a whole they represent up to 10% of the genome (for example, 8% in *Arabidopsis* [75]). They have not been resolved by any sequencing technology.
- **Satellites:** These are arrays of many tens or even thousands of identical or nearly identical copies of a repeated unit. They are abundant at centromeres and constitutive heterochromatin. For example, a total of 3% of the *Arabidopsis* genome consists of the 180 bp centromeric repeat [76]. As a result of microsatellites, most sequenced chromosomes are split into two sequences, the right arm and the left arm, since the repetitive, centromeric sequence is unknown.
- **Microsatellites or SSRs (simple sequence repeats):** These are short tandem repeats (in the range of kbp) of short motifs (1–5 bp) repeated a few hundred times or less, with different microsatellites having different motifs. They are often highly polymorphic with regard to the number of repeat units in a repeat [77]. Microsatellites are mainly located at the subtelomeric region that forms a border between distally positioned structural genes and telomeres, but they can also be found elsewhere.
- **Telomeric sequences:** These consist of a short repeat of a sequence motif similar to TTTAGGG in tandem arrays many hundreds of units long at the physical end of each chromosome arm. The number of telomeric repeats is a species-specific characteristic ranging from 2–5 kbp in *Arabidopsis* to 60–160 kbp in tobacco [62]. Moreover, the number of copies of the repeat motif also differs among the chromosome arms for the same genome, and may even vary from cell to cell and tissue to tissue [78]. They are usually still unknown at the sequence level in most species sequenced to date since they are nearly impossible to assemble.

## 4. Confounding Factors for Plant Genome Assembly

The apparent disconnection between the limitations of sequencing technologies (several hundreds of base pairs per read in the better cases) and their successful application to genome projects (several hundreds of megabase pairs for small-genome plants) can be explained by the clever combination of sequencing and computation. The resulting reads of a sequencing run must be combined into a reconstruction of the original genome using a computer program called ‘assembler’. The assembler tries to construct a ‘superstring’ that contains all reads as ‘substrings’. It must be understood that different assemblers are needed for *de novo* genome assembly, transcriptome assembly, or genome resequencing (the different rationales for assemblers are beyond the scope of this article), so no assembler is suitable for all approaches. Assembly and analysis of raw sequence data requires substantial bioinformatic effort and expertise [79]. In spite of the fact that different sequencing goals will require different assemblers, the confounding factors emerging from the nature of plant genomes, which are discussed in the following sections, complicate any assembly of plant reads.

### 4.1. Repetitive Nature of Plant Genomes

Most of the challenging features of plant genomes discussed above produce some kind of repeats in DNA. Repeat sequences are difficult to assemble since high-identity reads could come from different portions of the genome, generating gaps, ambiguities and collapses in alignment and assembly, which, in turn, can produce biases and errors when interpreting results. Simply ignoring repeats is not an option, as this creates problems of its own and may mean that important biological phenomena are missed [50]. Repeats would be easily resolved if a single read could span a repeat instance with sufficient unique sequence on either side of the repeat. But repeats longer than the read length specifically create gaps in the assembly and can only be resolved if there are paired-ends that span the repeat instance. Nearly identical tandem repeats (>97% identity) are often collapsed into fewer copies, and it is difficult for an assembler to determine the true copy number since genomic regions that share the same repeats can be indistinguishable, especially if the repeats are longer than the reads [50]. Inexact repeats (<95% identity) can be separated using high-stringency parameters. Repeats were not so critical in Sanger sequencing in which misassemblies and collapses occurred for only ~8% of the genome when duplications or repeats exceeded 95% sequence identity. Consequently, it is expected that repeats longer than 800 bp will suffer from the read-length methodology, regardless of whether it is NGS or Sanger [33]. It can be speculated that NGS short reads will have less power to resolve genomic repeats and require higher coverages to increase the chance of spanning short repeats. As a consequence, the most recent genome assemblies are much more fragmented than assemblies from a few years ago [51]. 

Repeat separation is assisted by high coverage but confounded by high sequencing error frequency: error tolerance leads to false positive joints that can induce chimeric assemblies, and this becomes especially problematic with reads from inexact (polymorphic) repeats. As a result, depletion of repeated sequences in assemblies becomes acute when the sequence identity exceeds 85%, resulting in the loss of ~16% of the genome [33], or ~5% of the genome being misassembled or missing [5]. The presence of duplicated and repetitive sequences in introns (a frequent event for genes in regions with >50% repetitive content) complicates complete gene assembly and annotation, leading to genes being broken among multiple sequence scaffolds: the more repetitive the region, the more scaffolds are obtained for the gene. After an assembly, nearly 70% of the genes are usually contained in single scaffolds [33], although exon shuffling is an artifact present in ~0.2% of those genes. 

The current and most robust methods for overcoming the repeat issues when assembling shotgun reads are: (1) increasing the read length (in fact, nowadays, a compromise solution is to combine short reads with long reads), (2) producing paired-end reads longer than the repeated regions [12], and (3) correlating contigs with genetic maps and/or FISH. This can be easily seen with recently assembled potato [60], tomato [80] and melon [81] genomes. In conclusion, the day that sequencing platforms generate error-free reads at high coverage and assembly software can operate at 100% stringency, repeats would be resolved and a single superstring solution would be obtained. However, advances in the newer technologies based on single-molecule sequencing are giving longer reads (2,000–5,000 bp by now), which will clearly help in the resolution of long repetitive DNAs.

### 4.2. DNA Contamination

Plant nuclear DNA extractions are always contaminated with mitochondrial and chloroplast DNAs that can confound further assemblies since there always are homologous genes between organelle and nucleus DNA. Moreover, samples from, for example, plant roots where the rhizosphere is not easily removed, are usually highly contaminated with cells from other organisms; and these contaminating cells contain their own DNA, which is usually not of interest in the sequencing goals. Also, contamination can be introduced during laboratory manipulation (adaptors, vector, linkers, poly-A, *etc.*). Unfortunately contamination is especially difficult to discern when sequencing is based on short reads. In fact, it has been found that contaminating sequences are usually present in the targeted, species-specific sequences, mainly in those that do not match with any homologous sequence in databases [33]. Therefore, in order to obtain a reliable assembly of genomes or transcriptomes, any possible contamination or artifact-prone sequence must be removed with pre-processing software (better than manual or in-house scripting methods), such as SeqTrimNext [82] (an evolution of SeqTrim fully prepared for NGS [83]). It must be taken into account, particularly in the case of genome assembly, that the phrase ‘garbage in, garbage out’ holds 100%, and that it can even be converted to ‘garbage in, nothing out’. Reads devoid of any contamination are less cumbersome to assemble and less prone to misassembling, and produce more reliable consensus [84].

### 4.3. Sequencing Errors

If sequencing datasets were completely error-free, every read (substring) should be contained within a superstring. But real biological sequences are more complicated since error rates may be as high as 1–4% per nucleotide, implying that many reads contain mismatches with respect to the solution superstring [85]. For example, it has been reported that the Illumina sequencers result in sequence-specific miscalls, GC biased errors [86,87], and more substitution-type miscalls than indel-type miscalls [88]. Roche/454 sequencers produce more indel-type miscalls than substitution-type miscalls due to well-known homopolymer length inaccuracy concerns [89]. The newer technologies based on single-molecule sequencing have been reported to have a 5–15% error rate [90]. Error frequencies can explain the sequence coverage variability and the unfavorable bias observed in reads [91]. In practice, tolerance for sequencing errors makes it difficult to resolve a wide range of genomic phenomena, ranging from polymorphisms to paralogues.

### 4.4. Read Length

Shorter reads are inherent to NGS technologies and deliver less information per read, thus confounding the computational problem of assembly by hindering the detection of contamination, repeats or polymorphisms/errors. Short reads cannot be assembled using any typical overlap-layout-consensus algorithm [92] because the repetitive sequences are usually longer than the reads, so many reads cannot be unambiguously assigned, resulting in very short sequence contigs. This prompted the development of new bioinformatic approaches such as de Bruijn graphs combined with Eulerian paths [93,94], and the over-sampling of the target genome from random positions. Assemblies constructed from short-read datasets are highly fragmented and require long reads to increase their contiguity [60,80]. The assemblers mostly recommended for short reads are ALLPATHS-LG, SOAPdenovo and SGA, each one with its own pros and cons with respect to assembly length and consensus errors [95]. The advent of technologies based on single-molecule sequencing are now giving reads of 2,000–5,000 bp in length [90], which could simplify the assembling process in the near future.

### 4.5. Quality Values

The quality value (QV) of each called base was widely used for Sanger sequences assembling [96]. Since its use greatly increases CPU and RAM requirements, QVs are used only by a small set of NGS assemblers [92]. Consequently, to save time and computational resources, most current assemblers assume that base calls are reliable. The presence of low-quality reads will reduce the effective coverage and obscure true overlaps between sequencing reads, thus fragmenting the assembly and risking the collapse of more repeats. This reinforces the need for a good pre-processing of NGS reads (e.g., using SeqTrimNext as explained before) to discard low QV fragments before assembly in order to avoid the assembling of inexistent sequences. For example, a 30 Gbp file of mate-pairs from HiSeq2000 could not be assembled within one week due to the presence of low quality nucleotides in the sequencer output; but this assembly was finished in four days in the same mainframe when reads were filtered for QV20 nucleotides [97].

### 4.6. Number of Reads and Coverage

Assembly is confounded by locations in which there are not enough overlapping reads to extend the sequence with confidence. It is easy to deduce that shorter read lengths will produce a larger number of gaps. The Lander-Waterman model offers a theoretical prediction of the minimum coverage needed to assemble large contigs depending on the read length [98]. For example, a three-fold (3×) coverage is sufficient when using Sanger technology, but a minimum of 15× coverage is required to assemble 100 bp reads into large contigs. However, considering the challenges depicted in the previous section, a minimum coverage of 7×–10× can work for Sanger technology, while 80×–100× is recommended in practice for short reads [32,33]. This high coverage will not resolve the concern about repeats but it is required to compensate the effective shorter length and sequencing errors of NGS technologies, which increase assembly complexity and intensify computational issues related to large datasets.

Short-read NGS technologies nowadays provide terabyte-sized data files, so coverage does not seem to be an issue, and previously intractable plant genomes (for example, pine genomes, which are seven- to 10-fold longer than the human genome and probably contain >95% repetitive sequences) are now feasible, at least in theory. Variation in coverage is introduced by chance, by variation of the copy number within DNA (*i.e.*, repeats), and by the technology *per se*. But when coverage is homogeneous along the genome, local biases can be interpreted as follows: Gaps are a consequence of very low coverage, and high-coverage is a diagnosis of an over-collapsed repeat. Unfortunately, coverage variability is the rule and undermines the coverage-based diagnostics. It can be speculated that the sequencing itself needs to be improved to reduce the biases, for example from GC composition and PCR, so that the coverage along the genome will be uniform and complete [99]. 

The overwhelming throughput of NGS raises a collateral issue related to data overload on a laboratory, institutional and community scale. In fact, the infrastructure costs for data storage, processing and handling are becoming more worrying than the costs of generating the reads. Since sequencing throughput is expected to increase in coming years, data storage and handling are becoming a real concern [14]. A more critical issue is computation: The comparison of each read with others required by the overlap-layout-consensus algorithms as well as the resolution of the Eulerian paths for de Bruijn graphs are the most time-consuming part of the assembling process. Therefore, the task could become never-ending or result in a faulty execution when temporary data do not fit in available RAM. The situation could arise that the right data and the right algorithm are available, but the right computer or software to hold calculations and memory are not. The most recent assemblers are focused on distributing among CPUs the processing load that cannot be managed with current serial algorithms. The de Bruijn graphs methods for assembly have the advantage of avoiding the all‑*versus*-all comparisons, but their use is limited when there are too many errors or there is too low coverage, since they lead to infinite loops in the Eulerian paths that produce erroneous ‘superstrings’ [100]. In conclusion, the type of choices to be made for plant sequencing using NGS remain the same: The importance of assembly size should be balanced against the cost of sequencing, the bioinformatics resources available, and the time the research team has to devote to the project (as in Heisenberg’s uncertainty principle, less costs and time in sequencing, more costs and time in assembling).

## 5. Seeking for the Best Assembly

When discussing plant genome assembly, it is important to distinguish between *de novo* approaches (where the aim is to reconstruct a new genome or transcriptome) and comparative approaches (referred to as mapping since the assembly uses a genome or transcriptome reference, or both). Mathematically, *de novo* assembly is such a difficult problem that, as yet, there is no efficient computational solution; in contrast, mapping is a much easier task. Neither approach is exclusive since after resequencing (mapping), there are always regions that differ significantly from the reference that can only be reconstructed through *de novo* assembly. Since *de novo* assemblies constructed from NGS technologies are highly fragmented, it has been proposed that a good genome assembly would have N50_contigs_ > 30 kbp, N50_scaffolds_ > 250 kbp, N50_super-scaffolds_ > 1 Mbp, >90% of the genes represented (as measured by previous transcriptomics analyses), and >90% coverage of full-length cDNAs [14]. For now, it should be evident that the ability to assemble plant genome data is constrained by the absence of bioinformatics tools designed to cope with the challenging features present in all plant genomes. Hence, genome assembly is far from being a resolved problem, and the worst consequence is the probably unexpected, artifactual explosion of apparent lineage-specific genes leading to gross incongruities [1]. It is a fact that different transcriptomics projects contain 20–40% unigenes that do not have an orthologue in another plant (e.g., [41,42]). Besides the species-specific genes, the most part of these unigenes may represent ‘garbage sequences’ generated by errors within the amplification and/or sequencing technology. The percentage of this garbage will be known more precisely as more and more transcriptomes and genomes are reported. In the meantime, we have developed the bioinformatics tool Full-LengtherNext [101] that can inform which unigenes may be garbage or putative species-specific unigenes [66].

Many assemblers designed to handle Sanger reads were found to be impractical when dealing with NGS data. The response was to develop new assemblers employing qualitatively new approaches that seemed to be appropriate for assembly from human to *Arabidopsis* genomes (to cite a few, CABOG, Newbler, ABySS, SOAPdenovo or ALLPATHS), although their true success depends largely on the sophistication of their heuristics for real reads to solve the existing issues [12]. They generally require servers or clusters with >500 gigabytes of RAM and many terabytes of available disk space. The decrease in cost of servers, the emergence of supercomputing centers, and the development of cloud computing, mean that they are available at a negligible cost. But new sequencing projects such as loblolly pine [102] or maritime pine [103] with 22–30 Gbp genomes, are increasing the computational demands by nearly another order of magnitude, and no proven technology is available to resolve this assembly. Assembler performance was evaluated last year in a competitive framework with both simulated and real datasets of small, simple genomes. Results confirmed that the final sequences were highly dependent on the assembler and pipeline used [95], although it can be said that assemblers for long reads produce longer contigs and scaffolds with more indels and underrepresentation of repeats, while the de Bruijn-based assemblers include shorter contigs and scaffolds, more mismatches and the highest representation for repeat regions [34]. Most assemblies nowadays rely on one single assembler, but as different assemblers use different underlying algorithms, combining different optimal assemblies from different programs can give a more credible final assembly [104]. The combination usually increased the N50 and median contig size, mapped more original reads, and diminished the final number of contigs/scaffolds. This strategy is currently used for transcriptomes, and CAP3 [96] or Minimus [105] are good candidates for the second assembly process [106,107,108]. In the case of genome assembly, mammalian genomes have recently been assembled using this combined strategy [109], running SOAPdenovo and ABySS separately, and then combining the assembly with GAP5 to generate the final consensus sequences.

As the choices made at the beginning of any study will determine the degree of success of the sequencing project, it can be concluded that there is a strong need to develop plant-specific assemblers that can overcome the challenges of these genomes; moreover, new software should expend efforts in producing user-friendly interfaces since most bioinformatics projects are developing software tailored to their needs, which leads to the same software being reinvented over and over again by different research groups [79]. Researchers have to decide which plant genome will be sequenced, which NGS technology will be applied, and which assembling approach should be used. In Plantagora [34], researchers can find a substantial body of information for comparing different approaches to sequencing a plant genome, providing a platform of metrics and tools for studying the process of sequencing and assembling that can aid in the critical decision-making required for planning a plant‑sequencing project.

## 6. Concluding Remarks

Plant genome sequencing is a long way away from automatic sequencing and assembly providing a completely finished genome at low cost. At the moment, we are able to afford the reconstruction of complex plant genomes into highly useful drafts. The need remains for an assembler that can deal with the plant genome features that challenge sequencing and assembly, *i.e.*, mainly large, repetitive genomes; moreover, incremental algorithms that can update the assembly as new data become available are also desirable. To circumvent the bioinformatics bottleneck in the near future, efforts should be invested in (1) parallelization of the assembly process, which has been shyly approached with ABySS [110] and ALLPATHS-LG [109]; (2) processing speed and storage capacity of computers; and (3) developing a new sequencing platform that can provide longer reads with unbiased coverage that can overcome the complex repeats. This last point refers to the so-called third‑generation sequencing based on single-molecule sequencing, which is very promising with reads of 2,000–5,000 nt [90]. However, these technologies are relatively immature for immediate widespread application to plant genomes since to date an error rate of 5–15% has been reported.
